# Efficacy and safety of the artificial pancreas in the paediatric population with type 1 diabetes

**DOI:** 10.1186/s12967-018-1558-8

**Published:** 2018-06-28

**Authors:** Susanna Esposito, Elisa Santi, Giulia Mancini, Francesco Rogari, Giorgia Tascini, Giada Toni, Alberto Argentiero, Maria Giulia Berioli

**Affiliations:** Pediatric Clinic, Department of Surgical and Biomedical Sciences, Università degli Studi di Perugia, Piazza Menghini 1, 06129 Perugia, Italy

**Keywords:** Artificial pancreas, Closed-loop system, Glycated haemoglobin, Sensor-augmented pumps, Type 1 diabetes

## Abstract

**Background:**

Type 1 diabetes (DM1) is one of the most common chronic diseases in childhood and requires life-long insulin therapy and continuous health care support. An artificial pancreas (AP) or closed-loop system (CLS) have been developed with the aim of improving metabolic control without increasing the risk of hypoglycaemia in patients with DM1. As the impact of APs have been studied mainly in adults, the aim of this review is to evaluate the efficacy and safety of the AP in the paediatric population with DM1.

**Main body:**

The real advantage of a CLS compared to last-generation sensor-augmented pumps is the gradual modulation of basal insulin infusion in response to glycaemic variations (towards both hyperglycaemia and hypoglycaemia), which has the aim of improving the proportion of time spent in the target glucose range and reducing the mean glucose level without increasing the risk of hypoglycaemia. Some recent studies demonstrated that also in children and adolescents an AP is able to reduce the frequency of hypoglycaemic events, an important limiting factor in reaching good metabolic control, particularly overnight. However, the advantages of the AP in reducing hyperglycaemia, increasing the time spent in the target glycaemic range and thus reducing glycated haemoglobin are less clear and require more clinical trials in the paediatric population, in particular in younger children.

**Conclusions:**

Although the first results from bi-hormonal CLS are promising, long-term, head-to-head studies will have to prove their superiority over insulin-only approaches. More technological progress, the availability of more fast-acting insulin, further developments of algorithms that could improve glycaemic control after meals and physical activity are the most important challenges in reaching an optimal metabolic control with the use of the AP in children and adolescents.

## Background

Type 1 diabetes (DM1) is one of the most common chronic diseases in childhood and requires life-long insulin therapy and continuous health care support. To avoid long-term adverse effects of chronically elevated glucose, patients with DM1 should maintain glycaemic levels as near as possible to the physiological range [1, 2]. Glucose variability has been recently identified as a risk factor for morbidity and mortality in patients with DM1 [3]. The International Society for Pediatric and Adolescent Diabetes (ISPAD) guidelines have recommended a level of glycated haemoglobin (HbA1c) < 7.5% as an indicator of good metabolic control [4]. To reach this goal, continuous subcutaneous insulin infusion (CSII) combined with continuous glucose monitoring (CGM) is the therapy of choice, especially in young children [5].

In the last few years, two functions that prevent hypoglycaemia by interrupting insulin delivery have been created: the low glucose suspend (LGS) and predictive low glucose suspend (PLGS) functions [6, 7]. However, despite the notable progress made, the aim of obtaining good metabolic control is still far from being reached in the majority of patients. One of the reasons is the persistent fear of hypoglycaemia, which leads patients or caregivers to keep blood glucose above target values, especially in younger children [8]. An artificial pancreas (AP) or closed-loop system (CLS) have been developed with the aim of improving metabolic control without increasing the risk of hypoglycaemia in patients with DM1. As the impact of APs has been studied mainly in adults, the aim of this review is to evaluate the efficacy and safety of the AP in the paediatric population with DM1.

## Composition of an artificial pancreas (AP)

An AP is able to automatically adjust insulin delivery rates depending on the interstitial glycaemic level. In the 1970s, the first prototypes of CLSs appeared and could be used only in supervised inpatient settings [9–11]. In the following years, in particular in the last decade, many technological advancements have been made so that today, many portable and sophisticated devices capable of improving glycaemic control are available. Recently, the first CLS was approved by the U.S. Food and Drug Administration (FDA) [12].

**Fig. 1:**
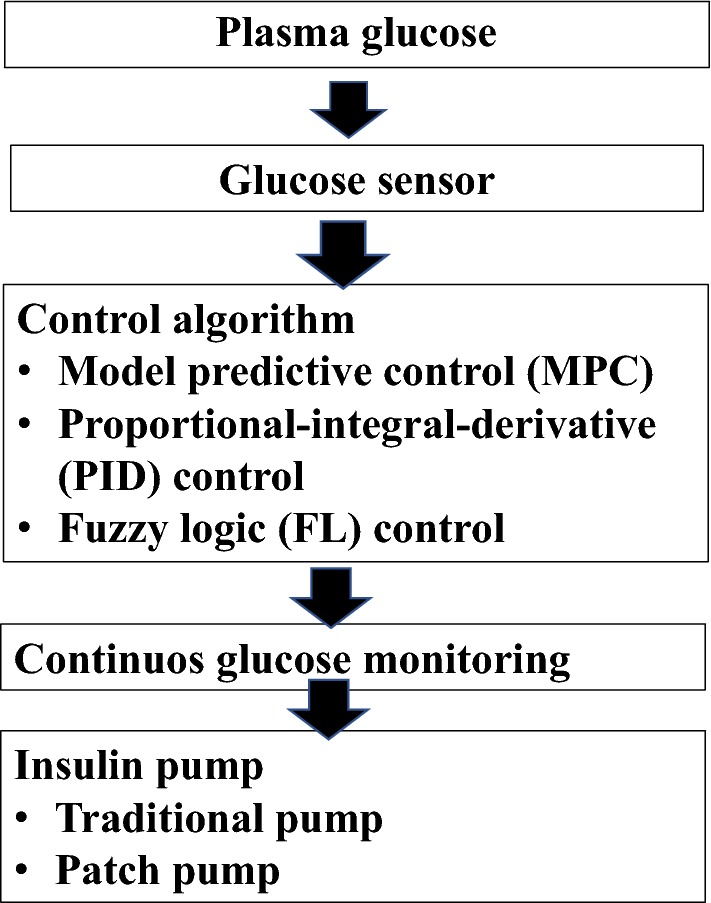
Composition of artificial pancreas (AP)

Figure 1 shows the composition of AP. An AP is composed of an insulin pump that delivers insulin to subcutaneous tissue and communicates with an interstitial glycaemic sensor. The insulin delivery rate is established by an algorithm that elaborates the sensor’s data. Concerning the infusion pump, two subtypes are available: the traditional pumps, with a visible infusion set, and the patch pumps, which are directly adhered to the skin and contain the infusion set inside. The CGM is the component of an AP that detects the interstitial glucose level and provides regular input to the control algorithm. Most of the latest studies have used subcutaneous enzyme glucose sensors with a coupled transmitter for wireless data transmission. The accuracy of a CGM, often expressed as the mean absolute relative difference (MARD), is an important parameter that continues to improve. The latest CGM models have reached an MARD < 10% and have been approved by the FDA as a reference for insulin dosing [13]. Furthermore, patients’ caregivers now have the opportunity to receive alerts in case of hypoglycaemia and hyperglycaemia thanks to the connection of CGM systems to smartphones. Finally, the glucose control algorithm is the central core of an AP and is able to control the glycaemic level. Three major glucose control algorithms have been developed by many research groups [14]: model predictive control (MPC) [15, 16], proportional–integral–derivative (PID) [17, 18], and fuzzy logic (FL) control [14].

The CLSs that have been investigated differ in many aspects of glycaemic control management. Several clinical trials have studied a bionic AP using a bihormonal pump system including glucagon, in addition to insulin, to avoid hypoglycaemia, while other studies involved a single-hormone AP, which uses insulin only to lower blood glucose and needs the absorption of fast-acting carbohydrates to treat hypoglycaemia. Some devices are hybrid closed-loop systems (HCLSs) that can be used for 24 h a day or only overnight and require meal announcement by the patients, which consists of the carbohydrate amount or qualitative information (i.e., small, medium or large meal). Other devices, called fully automated CLSs, are able to automatically adjust insulin delivery and establish meal insulin doses without the intervention of the patient. However, few trials have investigated the efficacy of fully automated CLSs, and most of them involved a small number of patients.

## Bihormonal artificial pancreas (AP) in the paediatric population

Some clinical trials have demonstrated the efficacy and safety of both insulin-only [19–25] and bihormonal APs in adults [26–29]. However, there are still few studies on bihormonal AP in the paediatric population, in particular in younger children.

The first clinical trial that used a bihormonal (insulin and glucagon) AP was performed in 2010 by El Khatib et al. [30]. This study demonstrated the safety and efficacy of a bihormonal AP in six adult patients with DM1 who used the device for over 48 h [5]. In 2014, Russell et al. conducted a randomised 5-day crossover study involving a higher number of DM1 patients (20 adults and 32 adolescents) in whom the fully automated bihormonal AP was used in an outpatient setting [29]. Their data showed that an automated bihormonal AP significantly improved glycaemic control by reducing mean blood glucose, increasing the time spent in the target range and reducing hypoglycaemia in both adults and adolescents older than 12 years. The same authors recently reported similar results in 19 preadolescent children with the use of a fully automated bihormonal CLS for 5 days (intervention arm) compared to the use of an SAP for another 5 days (control arm) in two diabetes camps [31]. Furthermore, a recent diabetes camp study, conducted by Haidar et al. compared a bihormonal AP with an insulin-only AP and CSII, demonstrating better glycaemic control and a significant reduction of time spent in hypoglycaemia with the use of the bihormonal system [28].

Overall, the available studies demonstrate the safety and efficacy of the bihormonal AP in paediatric age patients, but a greater number of long-term studies are required to compare the efficacy of bihormonal CLSs with that of single-hormone CLSs. Unfortunately, adolescents are usually studied together with adults, and most of these studies did not distinguish the results in the two groups [32–37]. Some studies comparing HCLSs with sensor-augmented pumps (SAPs) considered adolescents and adults separately and reported that overnight use of a CLS improved time spent in the target glycaemic range and reduced the number of hypoglycaemic events in both groups, without significant differences between the two populations [25]. Other authors showed some differences between the two groups [29, 38]. Sharifi et al. conducted an at-home, randomized crossover study with 16 adults and 12 adolescents comparing an HCLS to an SAP with the LGS function and reported a reduction of overnight hypoglycaemia with the HCLS, which was more significant in adolescents; they also found an improvement in the percentage of time within the target range (72–144 mg/dL) only in adults [38]. The different results between Sharifi’s study and the other trials [25] could be explained by the different devices used in the control period (i.e., Sharifi’s study used an SAP with the LGS function vs an SAP without any threshold suspend function being used in the other trials), the different algorithms analysed and the better glycaemic values of patients before the study [38]. However, the differences found between adults and adolescents could also be explained by the specific characteristics of the adolescent population, such as higher glycaemic variability and insulin resistance, psychological and physiological changes typical of puberty [39] and different eating habits.

In conclusion, further studies are needed to confirm the efficacy and safety of bihormonal AP in the paediatric population. The management of DM1 in adolescents and children has some critical issues, in particular in preschool children, such as very low insulin requirements, unpredictable eating patterns and physical activity and more frequent hypoglycaemia, especially at night. These features must be considered in specific clinical trials in order to demonstrate the advantages of APs in these patients.

## Single-hormone artificial pancreas (AP) in the paediatric population

The first trials in paediatric patients investigating the impact of CLSs on glycaemia have been performed in hospital settings. Two inpatient overnight studies using a single-hormone AP for 2 days demonstrated the safety of closed loop systems in younger children [40, 41]. In the first trial, Dauber et al. studied ten subjects aged < 7 years in a randomized controlled cross-over study comparing a CLS with standard open loop pump therapy and demonstrated an improvement of overnight glycaemic control without increasing the incidence of hypoglycaemia with the use of the CLS [40]. In the second trial, Elleri et al. conducted a randomized study that evaluated the use of an HCLS with standard insulin or diluted insulin in children aged 3–6 years [41]. They reported good overnight glycaemic control (glucose was maintained between 70 and 145 mg/dL for 72% of the time with standard insulin and for 83% using diluted insulin) without any hypoglycaemic events requiring treatment [41].

The safety and feasibility of a single-hormone AP was also demonstrated in an outpatient setting, in a randomized cross-over camp study with 30 children (5–9 years old) conducted by Del Favero et al. The authors demonstrated a significant reduction of hypoglycaemic episodes during both day and night (time spent in hypoglycaemia: 6.7% with open loop vs 2.0% with CLS; p < 0.001), but it was associated with a higher mean glucose value with the CLS [42]. A single-hormone AP reduces hypoglycaemia in adolescents and children [34, 35, 38, 43, 44], but the real advantage of a CLS compared to last-generation SAPs with LGS and PLGS functions is the possible improvement of the proportion of time spent in the target glucose range, reducing both hyperglycaemia and hypoglycaemia.

The results regarding the benefits of a single-hormone AP on the proportion of time spent within the target range in paediatric patients are conflicting. Some authors have demonstrated the improvement of time within target values [25, 34–36, 44–51], while other studies do not demonstrate any improvement of glycaemic control [30, 37, 52] or showed only a reduction of hypoglycaemia without an increase in time spent in the target range [38, 42]. On the other hand, in 2015 Thabit et al. demonstrated, in a randomized cross-over study, that a single-hormone AP could be used in free-living conditions for 12 weeks overnight by 25 children and by 33 adults both day and night and that a CLS, compared with SAP therapy, could reduce hypoglycaemia and increase the time spent in the target range. Both populations had a lower mean glucose level with the single-hormone AP compared to the SAP [25].

## Limits and future challenges on artificial pancreas (AP)

Despite the progress made, there are some important limitations that have to be overcome to achieve the ideal AP. First, there is the latency (which is at least 10 min but may be more in particular situations) between the blood glucose values and interstitial glucose values detected by presently available subcutaneous CGM models [53]. The direct consequence is that a CLS starts the insulin adjustment too late based on glycaemic variation. Second, the fast-acting insulin available absorbs too slowly from subcutaneous tissue, has a delayed onset and has a too long glucose-lowering effect (up to 4–5 h), with an increased risk of late hypoglycaemia, in particular after a meal insulin bolus [53]. Another challenge of an AP is mealtime glycaemic control: if the CLS algorithm varies the insulin infusion only when blood glucose is rising, the glucose-lowering effect will begin too late and will not maintain the glycaemic value in the target range. In fact, an HCLS, which was used in the majority of the studies, requires a pre-meal insulin bolus by the patient, usually with the need for CHO counting to avoid postprandial hyperglycaemia. Even though an HCLS can improve glycaemic control, reduce the time in hypoglycaemia and improve preprandial glucose, patients spend generally 70–75% of the time in the target range [54], and postprandial glycaemic excursions represent one of the most important limitations to reaching optimal metabolic control in the remaining percentage of time [55].

A recent review by Gingras et al. discussed different methods to overcome the problem of postprandial glycaemic control using a fully automated CLS [55]. Some study groups have experimented with different strategies to reach the goal: Weinzimer et al. used a fully automated CLS with an additional small manual priming bolus of insulin 15 min before meals and demonstrated a reduction of postprandial glucose in 17 adolescents in comparison with a fully automated CLS only [56]. The same authors were the first to test the adjunction of pramlintide to insulin CLS, demonstrating a significant delay in the time to peak prandial blood glucose and a reduction of glycaemic excursions in 8 adolescents and young adults [57].

Another strategy to improve metabolic control and to reduce the burden of carbohydrate counting for patients has been developed by El-Khatib et al. who used an adaptive meal-priming bolus with a bi-hormonal CLS [27]. With this approach, the patient has to select only the meal size (i.e., typical, more than usual or less than usual), and the CLS automatically administers 75% of the average prandial insulin bolus provided for previous meals at the same time of day. In their randomized controlled trial, El-Khatib et al. demonstrated that adaptive meal-priming boluses could improve the mean glycaemic value without increasing time spent in hypoglycaemia both in adults and in adolescents [27]. Furthermore, Russell showed the efficacy of a bihormonal CLS with adaptive meal-priming boluses in outpatient studies involving both adults and children [29, 52].

Unplanned physical activity, particularly in children, is another challenging daily circumstance that hinders good metabolic control in patients using CLS therapy. Recently, Dovc et al. showed an improvement of time spent in the target range after unplanned physical activity in 20 children and adolescents with the use of an HCLS compared to a SAP. However, the small sample size, the short duration of the study and the supervised in-hospital setting represent the most limiting factors of this trial [58]. De Bock et al. conducted an in-clinic observational study in 8 adults and adolescents utilizing an HCLS that included insulin delivery limits [59]. The results demonstrated that the insulin limit strategy was effective in avoiding overnight and exercise-induced hypoglycaemia, even in the presence of an over-reading glucose sensor [59]. Another future prospect is to improve the devices with auxiliary technologies that can provide information about the movement of the patient, such as an accelerometer or a recorder of the heart rate [60, 61].

With regard to the bi-hormonal CLS, one of the major limiting factors for long-term use is the poor stability of currently available glucagon formulations and the need for daily replacement of freshly reconstituted glucagon [62, 63]. However, a more stable glucagon formulation has been developed and is in clinical testing [64, 65].

## Conclusions

The real advantage of a CLS compared to last-generation SAPs with LGS and PLGS functions is the gradual modulation of basal insulin infusion in response to glycaemic variations (towards both hyperglycaemia and hypoglycaemia), which has the aim of improving the proportion of time spent in the target glucose range and reducing the mean glucose level without increasing the risk of hypoglycaemia. Many studies showed the efficacy of insulin-only CLS devices in increasing the time spent in normal glycaemia and reducing hypoglycaemia and hyperglycaemia compared to SAPs in adults, but there are few studies in the paediatric population, in particular in young children.

However, some recent studies demonstrated that also in children and adolescents an AP is able to reduce the frequency of hypoglycaemic events, an important limiting factor in reaching good metabolic control, particularly overnight. The advantages of the AP in reducing hyperglycaemia, increasing the time spent in the target glycaemic range and thus reducing HbA1c are less clear and require more clinical trials in the paediatric population, in particular in younger children. Although the first results from bi-hormonal CLS are promising, long-term, head-to-head studies will have to prove their superiority over insulin-only approaches. More technological progress on CGS, the availability of more fast-acting insulin, further developments of algorithms that could improve glycaemic control after meals and physical activity are the most important challenges in reaching an optimal metabolic control with the use of the AP in children and adolescents.

## Abbreviations

AP: artificial pancreas
CGM: continuous glucose monitoring
CLS: closed-loop system
CSII: continuous subcutaneous insulin infusion
DM1: type 1 diabetes
FDA: Food and Drug Administration
FL: fuzzy logic
HbA1c: glycated haemoglobin
HCLs: hybrid closed-loop systems
ISPAD: International Society for Pediatric and Adolescent Diabetes
LGS: low glucose suspend
MARD: mean absolute relative difference
MPC: model predictive control
PID: proportional–integral–derivative
PLGS: predictive low glucose suspend
SAPs: sensor-augmented pumps
